# Impacts of humic substances, elevated temperature, and UVB radiation on bacterial communities of the marine sponge *Chondrilla* sp

**DOI:** 10.1093/femsec/fiae022

**Published:** 2024-02-16

**Authors:** Tamara M Stuij, Daniel F R Cleary, Rui J M Rocha, Ana R M Polónia, Davide A M Silva, Antonio Louvado, Nicole J de Voogd, Newton C M Gomes

**Affiliations:** Department of Biology and Centre for Environmental and Marine Studies (CESAM), University of Aveiro, Campus Universitário Santiago, 3810-193, Aveiro, Portugal; Department of Biology and Centre for Environmental and Marine Studies (CESAM), University of Aveiro, Campus Universitário Santiago, 3810-193, Aveiro, Portugal; Department of Biology and Centre for Environmental and Marine Studies (CESAM), University of Aveiro, Campus Universitário Santiago, 3810-193, Aveiro, Portugal; Department of Biology and Centre for Environmental and Marine Studies (CESAM), University of Aveiro, Campus Universitário Santiago, 3810-193, Aveiro, Portugal; Department of Biology and Centre for Environmental and Marine Studies (CESAM), University of Aveiro, Campus Universitário Santiago, 3810-193, Aveiro, Portugal; Department of Biology and Centre for Environmental and Marine Studies (CESAM), University of Aveiro, Campus Universitário Santiago, 3810-193, Aveiro, Portugal; Naturalis Biodiversity Center, Darwinweg 2, 2333 CR, Leiden, the Netherlands; Institute of Biology (IBL), Leiden University, Sylviusweg 72, 2333 BE, Leiden, the Netherlands; Department of Biology and Centre for Environmental and Marine Studies (CESAM), University of Aveiro, Campus Universitário Santiago, 3810-193, Aveiro, Portugal

**Keywords:** climate change, coral reefs, DOM, sponge microbiome, terrestrial organic matter

## Abstract

Sponges are abundant components of coral reefs known for their filtration capabilities and intricate interactions with microbes. They play a crucial role in maintaining the ecological balance of coral reefs. Humic substances (HS) affect bacterial communities across terrestrial, freshwater, and marine ecosystems. However, the specific effects of HS on sponge-associated microbial symbionts have largely been neglected. Here, we used a randomized-controlled microcosm setup to investigate the independent and interactive effects of HS, elevated temperature, and UVB radiation on bacterial communities associated with the sponge *Chondrilla* sp. Our results indicated the presence of a core bacterial community consisting of relatively abundant members, apparently resilient to the tested environmental perturbations, alongside a variable bacterial community. Elevated temperature positively affected the relative abundances of ASVs related to Planctomycetales and members of the families Pseudohongiellaceae and Hyphomonadaceae. HS increased the relative abundances of several ASVs potentially involved in recalcitrant organic matter degradation (e.g., the BD2-11 terrestrial group, Saccharimonadales, and SAR202 clade). There was no significant independent effect of UVB and there were no significant interactive effects of HS, heat, and UVB on bacterial diversity and composition. The significant, independent impact of HS on the composition of sponge bacterial communities suggests that alterations to HS inputs may have cascading effects on adjacent marine ecosystems.

## Teaser

Humic substances modified bacterial composition within *Chondrilla* sp. sponges and enriched bacteria potentially involved in recalcitrant organic matter degradation while temperature and exposure to UVB had only minor and negligible effects, respectively.

## Introduction

Sponges are abundant and speciose components of coral reef ecosystems, well known for their filtration capacity (Diaz and Rützler 2001, De Goeij et al. 2013). They host diverse associations of microbial communities, including viruses, archaea, fungi, protozoa, and bacteria (Taylor et al. 2007, He et al. 2014, Pascelli et al. 2018). Most marine bacteria remain uncultured with inferred roles based on genomics or compound-uptake experiments. These studies support the potential importance of bacterial symbionts for nutrient acquisition and defense mechanisms (Selvin et al. 2010, Moitinho-Silva et al. 2017a, Burgsdorf et al. 2022). In the high microbial abundance (HMA) sponge *Aplysina aerophoba*, e.g. microbes accounted for the majority (65%–87%) of its dissolved organic carbon assimilation (Rix et al. 2020). Autotrophic photosymbionts, e.g. Ca. *Synechococcus spongiarum*, of the Indo-Pacific sponge *Theonella swinhoei* (Burgsdorf et al. 2022), in turn, were shown to transfer their photosynthates to inner sponge layers (Burgsdorf et al. 2022). The bacterial contribution to host defense is inferred from their production of a wide variety of bioactive secondary metabolites (Mohan et al. 2016).

Several sponge species have shown relatively little change in bacterial composition across space and time (Erwin et al. 2015, Campana et al. 2021), although some other studies have found that the bacterial communities of a number of sponge species appear to be structured by spatial and environmental parameters (Olson et al. 2014, Busch et al. 2020, Cleary et al. 2022). Short-term heat exposure experiments, e.g., significantly impacted the bacterial composition of two sponge species: the common blue aquarium sponge, *Lendenfeldia chondrodes*, and the red excavating sponge *Rhopaloeides odorabile* (Webster et al. 2008, Fan et al. 2013, Vargas et al. 2021). In *R. odorabile*, this bacterial community alteration was accompanied by host-tissue deterioration (Webster et al. 2008, Fan et al. 2013), while in *L. chondrodes*, no tissue damage or bleaching was observed (Vargas et al. 2021). Periods of elevated temperatures are often accompanied by intense UVB radiation and have been linked to El Niño southern Oscillation events (Hughes et al. 2017). As these events are predicted to grow more frequent and severe (Ying et al. 2022), the future state of coral reef ecosystems will be determined by how they respond to these environmental disturbances. The interactive effects of elevated temperatures and UVB on sponge bacterial communities remain, however, less studied.

In addition to climatic factors, marine ecosystems have also been affected by changes to the quality and quantity of terrestrially derived organic inputs (Felgate et al. 2021, Curra-Sánchez et al. 2022). Terrestrially derived dissolved organic matter (tDOM) mainly enters the marine environment via underwater cave systems or river runoff and is found in greater concentrations near coastal ecosystems (Esham et al. 2000). Humic substances (HS) are a major component of tDOM; these are complex organic compounds mainly formed through the decomposition of plant organic matter in terrestrial ecosystems (MacCarthy 2001). In the water column, HS provide UV-protective properties to reef organisms by absorbing sunlight in the ultraviolet (UV) spectrum (280–320 nm) (Ferrier-Pagès et al. 2007, Ayoub et al. 2012, Sharpless et al. 2014). Moreover, HS have been shown to promote bacterial diversity and the growth of potentially beneficial bacteria in fish (Louvado et al. 2021), and favor the growth of particular bacterial groups within marine bacterioplankton communities (Nardi et al. 2021).

The quantity and quality of tDOM entering marine ecosystems has shifted due to changes at the terrestrial–aquatic interface. Conversion of natural wetlands and coastal forests to urbanized areas and agricultural fields has led to increased inputs of nutrients and pollutants, at the expense of natural inputs of natural organic substances including HS (Spaccini et al. 2006, dos Santos et al. 2019). Soils of deforested agricultural fields, e.g., produce 38%–53% less HS than natural forests soils (dos Santos et al. 2019). Although previous studies have highlighted the importance of DOM in structuring sponge microbial communities (De Goeij et al. 2013, Campana et al. 2021, Shore et al. 2021), the specific effects related to the reduction and modification of HS in adjacent marine ecosystems, and sponges in particular, are largely unknown.

In this study, we used an experimental life support system (ELSS) to investigate the independent and interactive effects of HS supplementation, elevated temperature, and UVB radiation on the bacterial communities associated with the tropical sponge *Chondrilla* sp. (Chondrillida: Chondrillidae). The results presented here are an in-depth analysis of an earlier study, which discussed multiple coral reef biotopes (Stuij et al. 2023b). Species of the genus *Chondrilla* are abundant on reefs in both tropical and temperate oceans (Usher et al. 2004). *Chondrilla* spp. have been shown to associate with a diverse array of microbial symbionts, including Cyanobacteria, Bacteriodetes, Acidobacteria, and Proteobacteria (Usher et al. 2001, Hill et al. 2006, Thiel et al. 2007). High microbial abundance (HMA) status has been suggested for *Chondrilla* spp. from the Caribbean and the Indo-Pacific, including *C. australiensis, C. caribensis*, and *C. nucula* (Usher et al. 2001, Hill et al. 2006, Alexander et al. 2014). With *Chondrilla* sp. as a model organism, we tested the hypothesis that the individual and interactive effects of HS, elevated temperature, and UVB will affect the composition and diversity of sponge bacterial communities.

## Methods

### ELSS design

The structure of the ELSS developed in this study was based on a microcosm system previously developed to assess the effects of global climate change and environmental contamination on sediment communities (Coelho et al. 2013). This system was modified and validated to allow the evaluation of the effects of HS, UVB radiation, and temperature on host organisms and their associated microbial communities under laboratory-controlled conditions (Stuij et al. 2023a,b). The ELSS included two frames of 16 glass aquaria each (referred to as microcosms, 23 cm in height, 16 cm length, and 12 cm width), which were individually connected to other aquaria (referred to as reservoirs, 30 cm in height, 12 cm length, and 12 cm width). Each reservoir–microcosm unit contained a functional water volume of ~5 l. The microcosms and reservoirs contained outflow-holes (two cm in diameter) positioned 3 cm and 5 cm below the top of the glass, respectively (Figure S1, Supporting Information). Water was circulated within microcosm–reservoir units using small hydraulic pumps, resulting in a constant flow rate of ~8.64 ± 0.66 ml s^−1^. Water temperature was regulated using water bath tanks, each surrounding four microcosms, equipped with aquarium heaters with an internal thermostat (V2Therm 100 Digital heater, 100 W). Constant aeration within microcosms was maintained using diffuser stones (1.5 cm in diameter, 3 cm in length) connected to an air pump (530 L/h, Resun) via small hoses. Lighting was controlled by four fully programmable luminaire systems (Reef—SET, Rees, Germany), each holding eight fluorescent lamps (Coelho et al. 2013). During the experiment, four UV fluorescent tubes (SolarRaptor, T5/54 W, Rees, Germany) and four full spectra fluorescent tubes (ATI AquaBlue Special, T5/54 W) were connected alternately and programmed to a 12-h diurnal light cycle, simulating photoperiod conditions of tropical latitudes. To block radiation in the UVB spectrum (290–320 nm), microcosms were covered randomly by transparent polyester films (Folanorm SF-AS, Folex coating, Köln, Germany). The film has been used in multiple studies, which evaluated UVB radiation (Müller et al. 2009, Rautenberger et al. 2013). In our experimental set-up, the film absorbed 90% of the UVB, 31% of UVA, and 9% of PAR irradiance. Detailed information on the light energy transmitted during the day can be found in Supplementary material 1 and Figure S2 (Supporting Information).

A multifactorial experiment was designed to test for the independent and interactive effects of HS supplementation, temperature and UVB radiation. Each factor had two levels, namely, HS supplementation (with versus without), temperature [normal (∼28 °C) versus heat (∼32 °C)] and UVB radiation (with versus without) for a total of eight conditions with four replicates each. The full experiment, thus, consisted of 32 microcosms. The temperature treatment was randomized in groups of four microcosms; HS supplementation, UVB radiation and the combination of both were randomly assigned within each group of microcosms with equal temperature. The eight conditions are abbreviated as follows: (1) *Control*, (2) *UVB*, (3) *Heat*, (4) *UVB + Heat*, (5) *HS*, (6) *UVB + HS*, (7) *HS + Heat*, and (8) *HS + UVB + Heat*.

Each microcosm was spiked with a coral reef sediment layer of ∼3 cm, consisting of a mixture of commercially available (Reef Pink dry aragonite sand, Red Sea) and natural coral reef sediment. The commercial sediment was washed and sterilized three times (autoclavation at 121ºC for 20 min; Otte et al. 2018) before use. Natural sediment was collected from a coral reef environment at a depth of 6 m in the Penghu Islands (Taiwan), south of Fongguei (22°19′50.5″N 120°22′19.8″E), and used unprocessed. Synthetic seawater was added to the systems, prepared by mixing coral reef salt (CORAL PRO SALT, Red Sea) with reverse osmosis grade water (V2Pure 360) to a concentration of 35 ppt. HS were added to half of the microcosms as follows: a concentrated HS stock solution (10 g l^−1^) was first prepared by dissolving commercially available HS (technical grade humic acid, Sigma-Aldrich) using NaOH in deionized water. Subsequently, the solution was neutralized to a pH of 8.0–8.2 by adding concentrated HCL. Total carbon (TC), inorganic carbon (IC), organic carbon (TOC: TC—IC), and nitrogen (N) in the concentrated HS stock solution were measured using IR detection on a Multi N/C TOC analyser (Analytica Jena; Table S1, Supporting Information). The TOC concentration in the stock solution equaled 45.82 ± 5.12 mg l^−1^. This concentrated stock solution was then used to enrich the synthetic seawater to a final HS concentration of 7.5 mg l^−1^. Derived from the stock solution measurements, this resulted in an addition of 2.83 ± 0.32 µmol TOC l^−1^. The concentration of TOC used in this experiment was in the range of terrestrial dissolved organic carbon (tDOC) previously detected by Zhou et al. (2021) and Kaushal et al. (2022) in multiyear biogeochemical time series analyses of shallow coral reef waters in the central Sunda Shelf (Singapore Strait).

A total of 20% of the water in the reservoir–microcosm unit was renewed each day by adding 1 l of freshly prepared synthetic seawater either with or without HS, to the respective microcosms. Stability of the HS concentration in the water column of the microcosms was monitored using UV spectrometry (Eaton 1995). The absorbance at a wavelength of 300 nm was relatively stable over the course of the experiment and averaged 0.017 ± 0.003 AU among microcosms. The water was heated to 28°C in all microcosms and the UVB absorbing films were applied to all microcosms.

After 8 days of initial system stabilization, we added one specimen of the sponge *Chondrilla* sp. to each microcosm. Alongside the sponge, we added one specimen of four other coral reef species into each microcosm. These included two hard corals, *Montipora digitata* (Dana, 1846) and *Montipora capricornis* (Veron 1985), one soft coral, *Sarcophyton glaucum* (Quoy & Gaimard, 1833) and one zoanthid, *Zoanthus* sp. The effects of HS, temperature, and UVB on these organisms and their microbial symbionts are discussed elsewhere (Stuij et al. 2023b). After introducing the organisms, the microcosms were maintained under the specified conditions for 21 days in order to acclimate the organisms to the microcosm conditions. All animals used in this study were obtained from the collection of marine invertebrates cultivated at ECOMARE (University of Aveiro, Portugal). ECOMARE holds validated coral reef culture systems (Rocha et al. 2015), which have previously been used to study environmental effects on coral reef species under experimentally controlled conditions (Rocha et al. 2020). These animals were cultivated at 26°C, a salinity of ∼ 35 ppt, and pH of ∼ 8.2 (Rocha et al. 2015).

**Figure 1.**
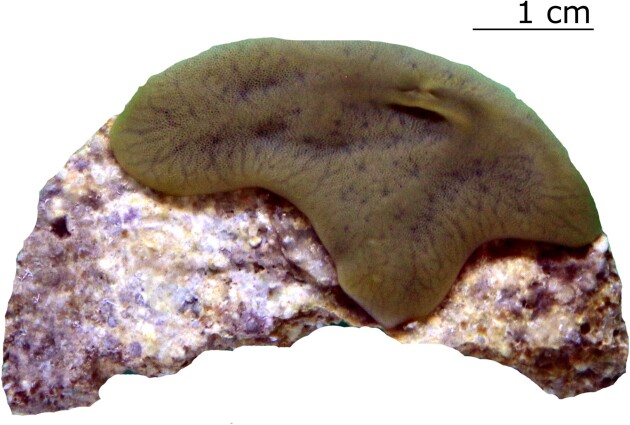
Fragment of *Chondrilla* sp. used in the present study.

The *Chondrilla* sp. analyzed in the present study grew on the live rocks present in the culture system collected from Indo-Pacific reefs. It is an encrusting sponge with a brown–olive green color and cartilaginous consistency (Fig. 1). Identification of the specimen to the genus *Chondrilla* (family Chondrillidae) was done by N.J. de Voogd. Species of the genus *Chondrilla* possess a relatively simple skeleton and few spicule types (Usher et al. 2004). So far, 16 valid *Chondrilla* species are known worldwide and four have been described from Indonesia, these are *Chondrilla australiensis* Carter, 1873, *Chondrilla grandistellata* Thiele, 1900, *Chondrilla jinensis* Hentschel, 1912, and *Chondrilla mixta* Schulze, 1877 (Putra et al. 2023), but neither of them fitted the characters of our species precisely. The outer morphology and skeletal features between species of *Chondrilla* are very similar and there are only a few characters that can be used to distinguish between species (Fromont et al. 2008). Given the above, we could not identify our specimen to species level and refer to the sponge studied here as *Chondrilla* sp.

We collected forty 1 cm^2^ pieces from larger mother colonies, each with a single osculum. We used a scalpel to remove these pieces and then securely attached them to carbonate stones using strong adhesive (100% cyanoacrylate). Following this, these fragments were given 4 months to heal and firmly adhere to the new surface before being moved to the microcosms for further study. Before fragmentation, we made sure the original sponge colony was healthy by observing its pumping activity with fluorescent dye. We verified the health of the sponge fragments by noting growth and attachment to the carbonate stones. A total of 32 cultivated sponges were haphazardly selected from the ECOMARE culture system and transplanted into the microcosms. Among the remaining cultivated sponges at ECOMARE, four were utilized to analyze the sponge-associated bacterial communities under original culturing conditions (non-transplanted sponges).

**Figure 2.**
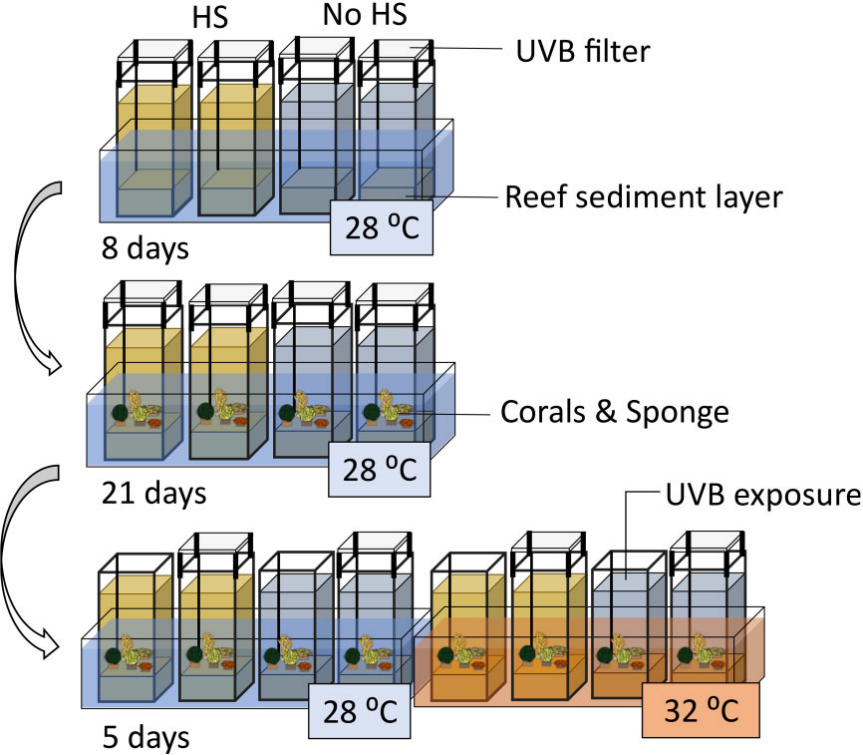
Graphical representation of the experimental set-up, previously displayed in Stuij et al. (2023a).

After the acclimatization period, heat and UVB treatments were applied for 5 days as follows. In the heat-treated microcosms, temperature was gradually increased over the course of 3 days from 28.0 ± 0.6°C at day 1 to 31.0 ± 0.6°C at day 3. In the UVB-treated microcosms, the UVB absorbing film was removed, which resulted in daily doses of 2.43 × 10 ^−2^ J cm^−2^ UVB radiation during 5 days. A graphical summary of the different phases of the experiment can be found in Fig. 2.

### Physical and chemical parameters

Water temperature, pH, dissolved oxygen, and salinity (Multi 3420 multimeter, WTW GmbH, Weilheim, Germany) were periodically monitored during the acclimatization period (one measurement per day) and experimental phase (two measurements per day). Water samples for determining dissolved inorganic nutrient concentrations (nitrate NO_3_^−^; nitrite NO_2_^−^ ammonium NH_4_^+^, and phosphate PO_4_^3−^) were taken every 7 days, using disposable syringes (50 ml), and were measured immediately, according to the Sali test kit protocol (colorimetric method test kit, salifert, Aquarium Masters). Inorganic nutrients (nitrate NO_3_^−^; nitrite NO_2_^−^; ammonium NH_4_^+^; phosphate PO_4_^3−^; and sulphate SO_4_^2−^) and total organic carbon (TOC) concentrations were analyzed in the sediment porewater after eight, 28 and 34 days. TOC and SO_4_^2−^ were measured in the sediment pore water to get insight into the build-up of organic matter and the biochemical regime in the sediment. These measurements were not taken from the water column as the conditions here are likely more variable and influenced by the daily water renewal. Sediment porewater samples were obtained using Rhizon flex samplers with a pore size of 0.6 µm (product number 19.60.25F, Rhizosphere Research Products) using 50 ml disposable syringes. Nitrate NO_3_^−^, nitrite NO_2_^−^, ammonium NH_4_^+^, and phosphate PO_4_^3−^ concentrations were analyzed using photometric methods following the standard analytical protocol of the Supelco Spectroquant® test kits 1.14942, 1.14776, 1.14752, and 1.14848, respectively (Merck). Immediately after collection, aliquots of the pore water samples were sent to an external service provider (A3lab) for sulphate SO_4_^2−^ and TOC analysis. SO_4_^2−^ was measured by turbidimetry using discrete spectrophotometry following the standard analytical method: CZ_SOP_D06_02_016 (SM 4500-SO4). TOC was measured using infrared (IR) detection following the standard analytical method: CZ_SOP_D06_02_056 (SM 5310).

### Bacterial community analysis

#### Sampling and DNA extraction

At the end of the experiment, all *Chondrilla* sp. specimens (32 sponges) were sampled from the microcosms. Upon collection, the sponges were photographed. These photos were used to estimate their size based on surface area in the software *Image J*. Four specimens were sampled from the ECOMARE facility to analyze the bacterial community composition of the sponges under their original culture conditions. All collected samples were washed with filtered and sterilized artificial seawater (with a pore size of 0.22 µm) and carefully cut off their carbonate stones using a scalpel and subsequently weighed. The wet weight of the specimens and size upon collection varied between 0.06–0.60 g and 1.92–5.99 cm^2^. Controls for ELLS contamination with environmental DNA and sample collection were performed as previously described (Stuij et al. 2023a). All samples were frozen at −80°C until DNA extraction. An overview of the metadata, size and weight of the samples can be found in Table S2 (Supporting Information).

PCR-ready genomic DNA was isolated using the FastDNA® SPIN soil Kit (MPbiomedicals) following the manufacturer’s instructions. Due to the small size of *Chondrilla* sp., whole organisms were used for extraction. Blank negative controls, in which no tissue was added to the Lysing Matrix E tubes, were also included. Microbial cell lysis was performed in the FastPrep Instrument (MP biomedicals) for 2 × 40 s at a speed setting of 6.0 ms^−1^. Extracted DNA was eluted in 50 µl of DNase/pyrogen-free water and stored at −20°C until further use.

#### 16S rRNA gene library preparation and sequencing

The V3–V4 variable region of the 16S rRNA gene was amplified using primers 341F 5′CCTACGGGNGGCWGCAG′3 and 785R 5′GACTACHVGGGTATCTAATCC′3 (Klindworth et al. 2013) with Illumina Nextera XT overhang adapters for a dual-PCR library preparation approach. PCRs were performed using 1–3 µl of DNA template, 10 µl of HS ReadyMix (KAPA HiFi Roche), and 0.6 µl of the forward and reverse primers in a concentration of 10 pMol µl^−1^. Reaction mixes were finalized by the addition of DNase free distilled water (Ultrapure, Thermoscientific) to a final volume of 20 µl. The PCR conditions consisted of initial denaturing at 95°C for 3 min, followed by 30 cycles of 98°C for 20 s, 57°C for 30 s, and 72°C for 30 s, after which a final elongation step at 72°C for 10 min was performed. We checked for the success of amplification, relative intensity of the bands, and contamination using 2% Invitrogen E-gels with 3 µl of PCR product.

PCR products were cleaned with magnetic beads at a ratio of 0.9:1 using a magnetic extractor stamp, after which a second PCR was performed. The 25 µl reaction mix consisted of 4 µl of the first PCR product, 12.5 µl HS ReadyMix (KAPA HiFi Roche), 2 × 1 µl (concentration of 10 pMol µl^−1^) MiSeq Nextera XT adapters (dual indexed, Illumina), and 6.5 µl of mQ water (Ultrapure). PCR conditions consisted of initial denaturing at 95°C for 3 min, followed by eight cycles of 98°C for 20 s, 55°C for 30 s, and 72°C for 30 s, after which a final elongation step at 72°C for 5 min was performed. DNA molarity and fragment sizes of the resulted PCR products were measured on a fragment analyzer 5300 (Agilent) and subsequently normalized and pooled together using the Qiagen QIAgility. The pool of normalized DNA was cleaned one last time using magnetic beads at a ratio of 0.65:1 and thereafter sequenced at a commercial company (Baseclear, Leiden, the Netherlands) on an Illumina MiSeq platform using 2 × 300 bp paired-end sequencing (Illumina MiSeq PE300). Three negative control samples were included to detect possible contamination during library preparation and sequencing. Sequences from each end were paired following Q25 quality trimming and removal of short reads (< 150 bp). The DNA sequences generated in this study can be downloaded from NCBI BioProject Id: PRJNA904682. Sample metadata, Biosample IDs and SRA numbers of the samples used in the current study are listed in Table S2 (Supporting Information).

### Sequencing analysis

Demultiplexed gzipped FASTQ files, which contained paired forward and reverse reads for each sample, were imported and visualized using QIIME2 (Bolyen et al. 2019). Subsequently, forward and reversed sequences were truncated to a length of 245 nt and 200 nt, respectively, using the DADA2 plugin (Callahan et al. 2016). The DADA2 analysis produced a quality filtered table of all amplicon sequence variants (ASVs), a fasta file of representative sequences, and a table summarizing the denoising statistics. Following this, the QIIME2 feature-classifier plugin with the extract-reads option was used to extract reads from the Silva database with the silva-138–99-seqs.qza file as input and the forward and reverse PCR primers as parameters. This produced a file of reference sequence reads, which was used as input for the feature-classifier plugin with the fit-classifier-naive-bayes option. ASVs generated by the DADA2 analysis were classified taxonomically using the feature-classifier plugin in QIIME2 with the classify-sklearn method. Mitochondria, chloroplasts, and Eukaryota were filtered out from the obtained ASV table using the QIIME2 taxa plugin with the filter-table method. The ASV count table is presented in Table S3 (Supporting Information).

Subsequently, we removed archaeal sequences, ASVs that were unassigned at the Domain and Phylum levels, in addition to ASVs, which occurred in the triple-autoclaved commercial sediment (control for sampling and eDNA contamination), and negative controls used for sequencing. ASVs removed following detection in the control samples are listed in Table S4 (Supporting Information). Overall, the removed ASVs were assigned to known contaminants, e.g., the genera *Ralstonia, Burkholderia–Caballeronia–Paraburkholderia, Reyranella, Bacillus*, and *Bradyrhizobium* (Salter et al. 2014, Glassing et al. 2016, Weyrich et al. 2019). Abundant ASVs were referenced against the NCBI nucleotide database using the NCBI Basic Local Alignment Search Tool (BLAST) (Zhang et al. 2000). BLAST identifies locally similar regions between sequences, compares sequences to extant databases and assesses the significance of matches.

### Statistical analysis

A table containing ASV counts was imported into R and used to analyse how transplantation and the treatments influenced bacterial composition and higher taxon abundance. Differences in rarefied richness (sample size of 15 556 sequences), evenness (calculated by dividing Shannon’s H’ by the number of ASVs in each sample), and higher taxon abundances between ECOMARE and *Control* samples, and among treatments were investigated using an analysis of deviance using the glm() function of the R package stats. A number of these variables included an excess of zero counts in the samples, therefore, we set the family argument to “tweedie” using the tweedie function in R with var.power=1.5 and link.power=0 (a compound Poisson–gamma distribution). Using the glm model, we tested for significant variation using the anova() function in R with the F test. To correct for multiple testing in the relative abundance of phyla, classes, and orders, we applied the Bonferroni correction. This correction accounts for type I errors and compares *P-*values to $\frac{\alpha }{n}$, in which α represents the threshold significance level and *n* the number of tests. For each taxonomic level, we analyzed if there was an effect in the four most abundant groups, resulting in four tests per taxonomic level and a corrected significance level of $\frac{{0.05}}{4} = 0.0125$. Variation in bacterial composition was visualized with principal coordinates analysis (PCoA). For the PCoA, the ASV table was rarefied to the minimum sample size (15 556 sequences) using the rrarefy() function of the R package vegan. For compositional analyses, the ASV table was log(*x* + 1) transformed (in order to normalize the distribution of data) and a distance matrix constructed using the Bray–Curtis index with the vegdist() function in the vegan package in R. Subsequently, we used the cmdscale() function of the R package stats with the Bray–Curtis transformed distance matrix as input. We tested for significant differences in ASV composition with a permutational analysis of variance (PERMANOVA) using the adonis2() function and for homogeneity of multivariate dispersion using the betadisper() function of the R package vegan (999 permutations). Detailed descriptions of the functions used here can be found in R and online in reference manuals of packages (e.g. http://cran.rproject.org/web/packages/vegan/index.html). For the factors that significantly explained variation in our dataset, we calculated the effect size (ω2) using the function adonis_OmegaSq(), with the adonis2 test result as input. The source code of this function is given in the Supplementary material. The function is based on the MicEco: adonis_OmegaSq() function of the package MicEco but adjusted so it works with adonis2.

To identify specific classes, orders and ASVs that associated with given treatments, we used a feature selection algorithm called “Boruta.” Boruta, named after a slavic forest demon, is a random forest wrapper, which is used to evaluate feature importance (Kursa et al. 2010). Boruta iteratively compares the importance of features with the importance of shadow features, created by shuffling the original ones. Features that have significantly less importance than shadow ones are consecutively dropped. On the other hand, features that are significantly better than shadows are identified as confirmed. It is assumed that confirmed features with the highest importance are most relevant to the outcome variable. In the present study, the Boruta() function from the Boruta package in R was used with HS and Heat as response variables and the relative abundance of bacterial classes, orders, and ASVs as features (predictive variables). The doTrace argument in the Boruta() function was set to 2 and the maxRuns argument set to 1000; other arguments used default values. Only ASVs with an absolute abundance of > 100 sequences across the dataset were included (58 ASVs). Significant predictor ASVs were referenced against the NCBI nucleotide database using NCBI BLAST (Zhang et al. 2000).

## Results

### Physical and chemical parameters

The results of the water quality analysis are summarized in Figures S3–S6 (Supporting Information). During the acclimatization phase, temperatures ranged from 26.4 ± 0.6°C in the morning to 27.6 ± 0.8°C in the afternoon. Salinity averaged 35.50 ± 0.74 ppt. pH varied from 8.10 ± 0.05 in the morning to 8.21 ± 0.07 in the afternoon for *HS*, and from 8.05 ± 0.06 to 8.16 ± 0.07 for *Control* microcosms. Dissolved oxygen levels ranged from 8.12 ± 0.21 mg l^−1^ in the morning to 8.39 ± 0.34 mg l^−1^ in the afternoon for *HS*, and from 8.15 ± 0.34 mg l^−1^ to 8.55 ± 0.44 mg l^−1^ for *Control* microcosms. During the experimental phase (see Figure S4, Supporting Information), microcosms without heat treatment had temperatures of 27.2 ± 0.5°C in the morning and 28.0 ± 0.6°C in the afternoon. In heat-treated microcosms, temperature increased gradually over 3 days from 28.0 ± 0.6°C on day 1 to 31.0 ± 0.6°C on day 3, averaging 31.4 ± 0.5°C and 31.2 ± 0.7°C on days 4 and 5, respectively. Dissolved oxygen levels ranged from 7.01 ± 0.40 mg l^−1^ in *Heat + UVB + HS* in the morning to 9.06 ± 0.36 mg l^−1^ in *UVB* microcosms in the afternoon. pH ranged from 8.09 ± 0.05 in *Heat* microcosms in the morning to 8.32 ± 0.024 in *UVB* microcosms in the afternoon.

During the experiment, NO_3_^−^, NO_2_^−^, NH_4_^+^, and PO_4_^3−^ concentrations in the water column and pore water remained below detection limits (< 3.23*10^−3^, < 0.22*10^−3^, < 8.32*10^−3^, and < 0.53*10^−3^ mmol l^−1^, respectively). Porewater SO_4_^2−^ concentrations showed a decreasing trend and mean values were 30.75 ± 0.34 mmol l^−1^ on day 8, 27.84 ± 1.15 mmol l^−1^ on day 28, and 26.75 ± 0.97 mmol l^−1^ on day 34. Independent effects of Heat and UVB, as well as an interactive effect between Heat and HS were observed (three-way ANOVA, Heat—F_1,23_ = 18.824, *P* < .001, UVB—F_1,23_ = 4.664, *P* = .041, Heat: HS—F_1,23_ = 7.421, *P* = .012). Sulphate concentrations in *Heat + HS* microcosms were significantly higher than in *HS* microcosms (Tukey HSD, Heat + HS: HS—*P*-adj < .001). Porewater TOC concentrations progressively rose throughout the experiment. No independent or interactive effect of our factors explained significant variation in the dataset (PERMANOVA using adonis2 in the R package vegan, *P* > .05). The mean values were 0.14 ± 0.01 mmol C l^−1^ on day 8, 0.25 ± 0.1 mmol C l^−1^ on day 28, and 0.41 ± 0.24 mmol C l^−1^ on day 34.

### The effect of transplantation on sponge bacterial communities

The bacterial composition and diversity of specimens obtained from the ECOMARE facility and their variance from the control specimens collected from the microcosms, are depicted in Figures S7 and S8 (Supporting Information). Bacterial richness and evenness did not differ significantly between ECOMARE and microcosm sponges (GLM, *P* > .05, Table S6, Supporting Information). Bacterial community composition, however, significantly differed between ECOMARE and microcosm sponges (PERMANOVA: F_1,6_ = 5.72, *R*^2^ = 0.489, *P* = .021, ω^2^ =0.43, PERMDISP: F_1,6_ = 1.26, *P* = .21). This variation was visualized in an ordination analysis (Figure S7b, Supporting Information) based on bacterial composition, which separated samples collected from ECOMARE and the microcosms on the first axis of variation. We subsequently investigated if variation occurred in higher taxonomic composition (four most abundant phyla, classes, and orders), and found some minor, non significant variance in the relative abundances of the four most abundant phyla and classes (see Figure S7c, Supporting Information). Of the four most abundant orders, HOC36 was significantly more abundant in ECOMARE samples than in the microcosm controls (GLM, *P* = .012; Table S6, Supporting Information). Sponges of both ECOMARE and the microcosms were dominated by the same group of ASVs (Figure S8, Supporting Information).

### Sponge bacterial communities under the independent and interactive effect of HS, heat, and UVB

After quality control and removal of ASVs assigned to chloroplasts and mitochondria, and ASVs unassigned at Domain and Phylum level, the dataset consisted of 875 721 sequences and 397 ASVs. A full overview of the observed phyla, classes and orders and their abundances is presented in Table S5 (Supporting Information). In terms of sequences, the most abundant phyla were Proteobacteria, Cyanobacteria, Acidobacteriota, Gemmatimonadota, Chloroflexi, and Actinobacteriota (all > 20 000 sequence reads). The phyla with the highest numbers of ASVs were Proteobacteria, Verrucomicrobiota, Bacteriodota, Planctomycetota, and Cyanobacteria (all > 20 ASVs).

### Bacterial diversity and composition

**Figure 3.**
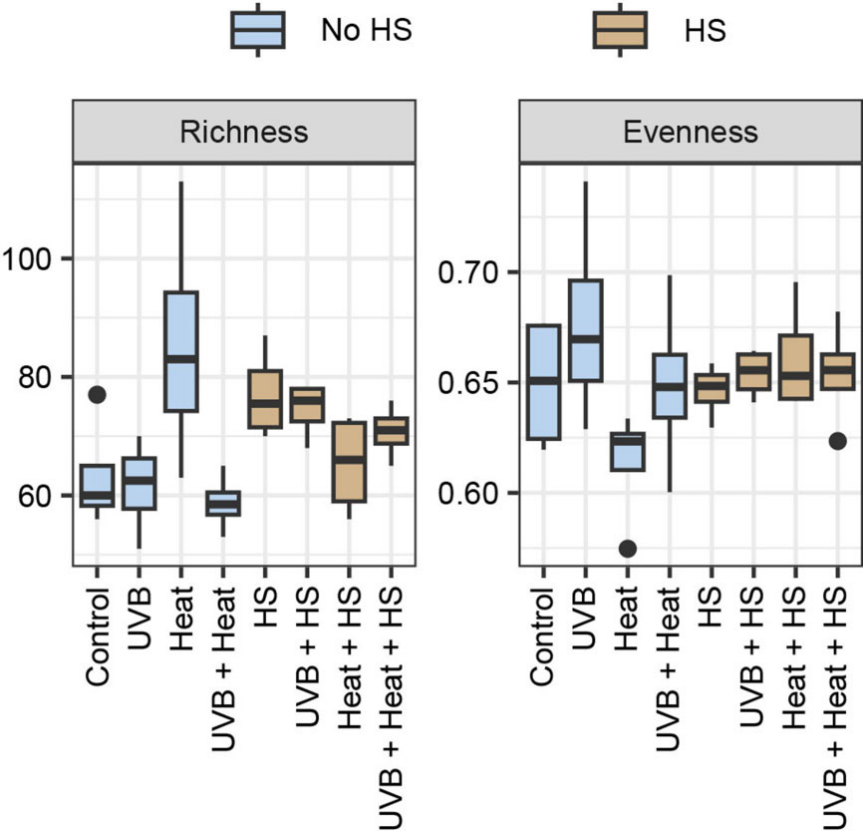
Boxplots of the rarefied richness and evenness under the independent and combined effects of UVB, heat, and HS supplementation. Diversity measures are grouped per treatment.

There were no significant differences (GLM, *P* > .0125) among treatments in any of the diversity indices. Bacterial richness varied from 58.96 ± 4.73 in *Heat + UVB* to 85.52 ± 19.43 in *Heat*. Evenness varied from 0.61 ± 0.03 in Heat to 0.68 ± 0.05 in *UVB* microcosms (Fig. 3; Table S6, Supporting Information).

**Figure 4.**
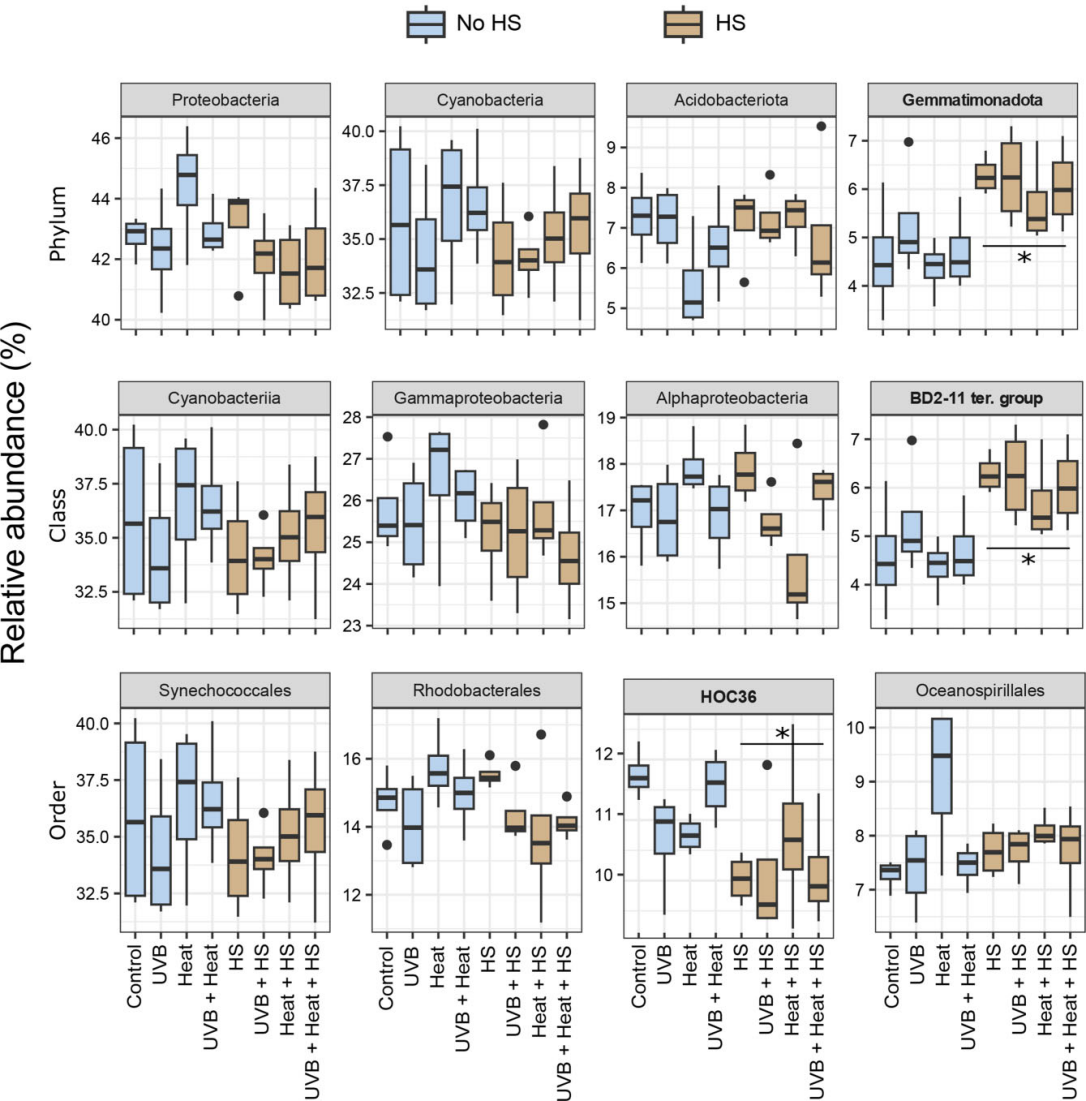
Boxplots of the relative abundance of the four most abundant phyla, classes, and orders under the independent and combined effects of UVB, heat, and HS supplementation. Relative abundances are grouped per treatment. Note that factor-related significant variation is indicated with an * (GLM, *P* < .0125). See Table S5 (Supporting Information) for detailed statistical results.

**Figure 5.**
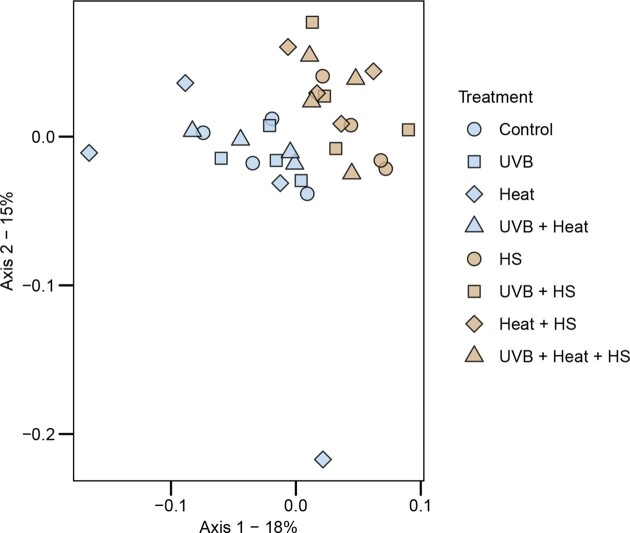
Ordinations showing the first two axes of the principal coordinates analysis (PCoA) of bacterial ASV composition. The PCoA was generated using the cmdscale() function in the R base package. Prior to the PCoA, the raw data were log(x + 1) transformed and used to produce a distance matrix based on the Bray–Curtis distance with the vegdist() function in the R-package vegan.

The relative abundances of the four most abundant phyla, classes, and orders are shown in Fig. 4. HS was a significant independent predictor of the relative abundances of the phylum Gemmatimonadota, the class BD2-11 terrestrial group and the order HOC 36 (GLM, *P* < .0125; Table S6, Supporting Information). The phylum Gemmatimonadota and class BD2-11 terrestrial group were more abundant in HS-supplemented microcosms, while the order HOC 36 was less abundant. HS (PERMANOVA: F_1,24_ = 4.848, *P* = .001, *R*^2^ = 0.136, ω^2^ = 0.10, PERMDISP: F_1,30_ = 0.01, *P* = .96) and Heat (PERMANOVA: F_1,24_ = 1.682, *P* = .026, *R*^2^ = 0.047, ω^2^ = 0.02, PERMDISP: F_1,30_ = 1.04, *P* = .35) were both significant independent predictors of bacterial community composition (Table 1). No independent effects of UVB or interactive effects of HS, Heat or UVB on bacterial community composition were observed. The PCoA presented in Fig. 5 shows that samples subjected to HS supplementation clearly separated along the first two axes of variation.

**Table 1.** Results of the PERMANOVA analysis. Coef: coefficient, Df: degrees of freedom, SumsOfSqs: sum of squares, and MeanSqs: mean squares. Significant test results are depicted in bold.

Df	SumsOfSqs	MeanSqs	*F*	*R* ^2^	*P*	Coef
1	0.023	0.023	1.682	0.047	**.026**	**Heat**
1	0.016	0.016	1.156	0.032	.253	UVB
1	0.066	0.066	4.848	0.136	**.001**	**HS**
1	0.01	0.01	0.739	0.021	.844	Heat:UVB
1	0.015	0.015	1.126	0.031	.285	Heat:HS
1	0.014	0.014	1.056	0.03	.376	UVB:HS
1	0.016	0.016	1.168	0.033	.249	Heat:UVB:HS
24	0.325	0.014		0.671		Residuals
31	0.484			1		Total

### Significant predictor higher taxa

**Figure 6.**
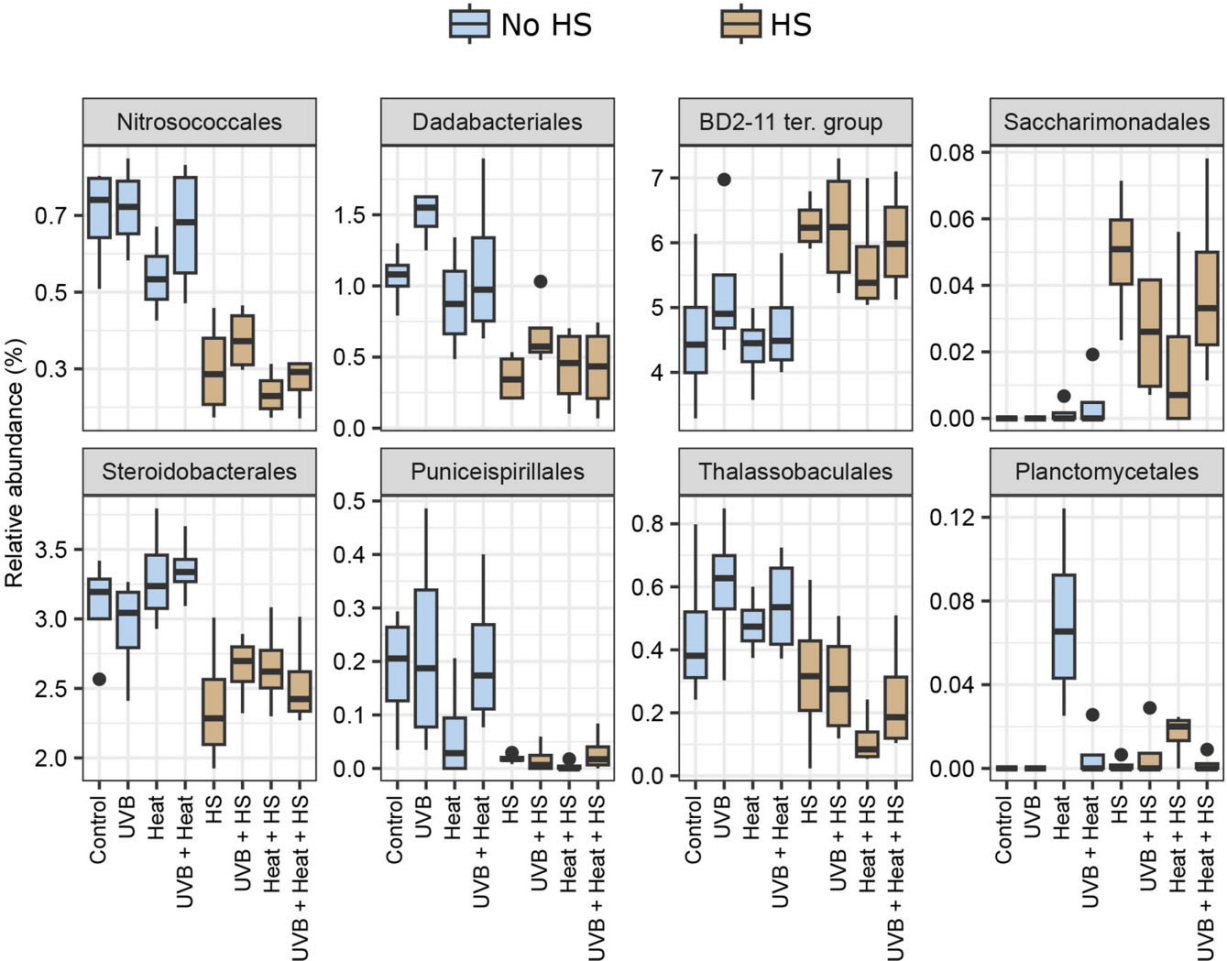
Boxplots of the relative abundance of the orders with more than 5.00% importance in the Boruta analysis with HS or Heat as response variable. Significant predictors for HS: Nitrosococcales, Dadabacteriales, BD2-11 terrestrial group, Saccharimonadales, Steroidobacterales, Puniceispirillales, and Thalassobaculales. Significant predictor for Heat: Planctomycetales. Relative abundances are grouped per treatment.

The Boruta analysis detected six classes and 11 orders as significant predictors of HS, whereas one class and two orders were found to be significant predictors of Heat (Table S7, Supporting Information). The relative abundances of the orders with the highest importance values (importance value > 5.00%) are presented in Fig. 6. Of these, the orders BD2-11 terrestrial group and Saccharimonadales were relatively more abundant and, the orders Nitrosococcales, Dadabacteriales, Steroidobacterales, Puniceispirillales, and Thalassobaculales relatively less abundant in HS-supplemented microcosms. The order Planctomycetales was the only significant and positive predictor of Heat with an importance value > 5.00% (Table S7, Supporting Information).

### Significant predictor ASVs

**Figure 7.**
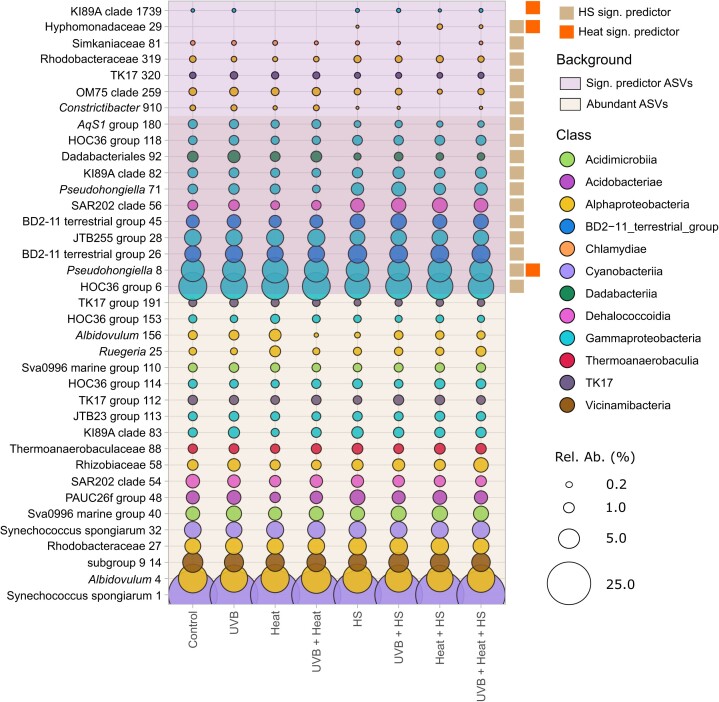
Mean relative abundances of the 30 most abundant and significant predictor ASVs (17 for HS; 3 for Heat). Relative abundances are grouped per treatment. Symbols are proportional to the relative abundance of the respective ASV and color-coded following their class-level taxonomic assignment. ASVs are labeled with their lowest taxonomic classification. Hs- and Heat-significant predictor ASVs are labeled at the right side of the figure.

Among the 30 most abundant ASVs, Boruta analysis detected 10 significant predictors of HS and one significant predictor of both HS and Heat (Fig. 7, Tables S7 and S8, Supporting Information). Among the less abundant ASVs, we detected five significant predictors of HS, one of Heat and one of both HS and Heat. Taken together, the abundances of these ASVs accounted for > 96% of all sequences.

Abundant ASVs, which were more abundant in HS-supplemented microcosms, were assigned to the BD2-11 terrestrial group (ASVs 26 and 45), the SAR202 clade (ASV-56), the KI89A clade (ASV-82), the HOC36 group (ASV-118), and the genus *Pseudochongiella* (ASV-71). ASVs, which were less abundant in HS-supplemented microcosms were assigned to the HOC36 group (ASV-6), the JTB255 marine benthic group within the family Woeseiaceae (ASV-28), the order Dadabacteriales (ASV-92), the genus *AqS1* within the family Nitrosococcaceae (ASV-180), and the genus *Pseudohongiella* (ASV-8) (Fig. 7).

Of the less abundant ASVs, ASVs 29 (Hyphomonadaceae) and 319 (Rhodobacteraceae) were more abundant, while ASVs 910 (Simkaniaceae), 259 (OM75 clade within Nisaeaceae), 320 (TK17 group), and 81 (*Constrictibacter*) were less abundant in HS-supplemented microcosms. For Heat, two ASVs (ASV-8, *Pseudohongiella*, and ASV-29, Hyphomonadaceae) were more, while ASV-1729, assigned to the gammaproteobacterial KI89A clade, was less abundant in heat-treated microcosms. The ASVs identified as significant predictors of HS and Heat were compared to sequences in the NCBI nucleotide database (Table S8, Supporting Information). A total of 14 of these had sequence similarities (ranging from 94% to 100%) to organisms previously detected in a variety of marine sponges, namely *Xestospongia muta, Ircinia variabilis, C. nucula, Ancorina alata, Ircinia strobilina, Plakortis halochondrioides, Aaptos aaptos, Svenzea zeai, Ircinia felix, Aplysina cauliformis*, and *I. strobilina*.

## Discussion

In this study, we used an ELSS to investigate the independent and interactive effects of elevated temperature, UVB radiation, and HS-supplementation on the bacterial communities of the tropical sponge *Chondrilla* sp. This model system allowed us to test hypotheses with respect to an impact of these factors on bacterial diversity and composition with a high level of experimental control and replication, which would be impossible to achieve with field studies alone.

### Physical and chemical conditions in the microcosms

Average values of water temperature, dissolved oxygen, pH, and salinity in the microcosms were similar to conditions measured at shallow coral reef sites (Guadayol et al. 2014, Bainbridge 2017, DeCarlo et al. 2017). During both the acclimatization (see also Stuij et al. 2023a) and experimental phase, we observed daily fluctuations in oxygen and pH (higher in the morning and lower in the afternoon). This variation is common at reef sites and is a result of net-photosynthesis during the day and respiration at night (Guadayol et al. 2014). During the experimental phase, oxygen levels were lower in heated microcosms, which can be attributed to the positive correlation between organismal metabolic rates and temperature (Brown et al. 2004). The effect of temperature was however marginal. The lowest values were observed in *Heat + UVB + HS* microcosms but still averaged 0.22 ± 0.013 mmol O_2_ l^−1^. The highest values were observed in *UVB* microcosms in the afternoon and averaged 0.28 ± 0.01 mmol O_2_ l^−1^. At reef sites, dissolved oxygen concentrations typically range from 50% air saturation to 200% air saturation (corresponding to 0.12–0.43 mmol O_2_ l^−1^ at 27°C), depending on location and time of day (Nelson and Altieri 2019).

Inorganic nutrients in the water column and sediment pore water (NO_3_^−^, NO_2_^−^, NH_4_^+^, and PO_4_^3−^) remained low (< 3.23*10^−3^ mmol l^−1^, < 0.22*10^−3^ mmol l^−1^, < 8.32*10^−3^ mmol l^−1^, and < 0.53*10^−3^ mmol l^−1^, respectively) throughout the experiment. Inorganic nutrient concentrations below these values were previously observed in Pacific coral reefs as well (Silbiger et al. 2018). Porewater sulphate concentrations of surface sediments have been observed to vary between 23.96 and 30.00 mmol SO_4_^2−^ l^−1^ (Alongi 1995, Werner et al. 2006). At the first time point, sulphate was relatively high (30.75 ± 0.34 mmol l^−1^), but fell to levels previously observed by Alongi (1995) and Werner et al. (2006) in coral reef environments. Although we detected relatively low TOC in the ELSS at the beginning of the experiment (0.14 ± 0.01 mmol C l^−1^), TOC concentrations increased to 0.41 ± 0.24 mmol C l^−1^ at day 34. These values were in the range of DOC concentrations (∼ 0.25 to 0.67 mmol C l^−1^) in surface sediment pore water at Great Barrier Reef sites (measured using 0.4 µm membrane filters; Lourey et al. 2001). Note that the membrane pore size of our samplers was 0.6 µm, which included a slightly larger fraction of total organic matter compared to the measurements taken at the Great Barrier Reef.

### The effect of transplantation on the bacterial communities of *Chondrilla* sp.

The transplantation of the sponges affected bacterial composition, indicating a certain level of microbial adaptation to the new environmental conditions. Sponges of both ECOMARE and the microcosms were dominated by the same group of ASVs (Figure S8, Supporting Information; see also Stuij et al. 2023a).

### The independent and interactive effect of HS, heat, and UVB on the bacterial communities of *Chondrilla* sp.

Our results revealed the presence of a core bacterial community, composed of highly prevalent ASVs across all host individuals and which remained relatively abundant irrespective of the environmental conditions. However, we also detected bacterial populations that appeared more responsive to the independent effects of HS and temperature, explaining 14% and 5% of the observed variation in community composition, respectively. In line with the presence of a relatively stable core community, Thiel et al. (2007) and Erwin et al. (2015) observed high host-specificity, but low seasonal and interannual compositional variation in bacterial communities of a number of HMA and LMA sponge species, including *C. nucula, Dysidea avara, Chondrosia reniformis, Agelas oroides, Axinella damicornis, Petrosia ficiformis*, and *Spirastrella cunctatrix*.

ASVs 1 and 4, assigned to Ca. *S. spongiarum*, and to *Albidovulum* sp., respectively, were stable core members. In congruence with our results, previous studies showed that members of the Ca. *S. spongiarum* group remained abundant in sponge hosts (*Iricinia variabilis* and *Xestospongia muta*) across a range of temperatures and different irradiance regimes (Erwin et al. 2012, Lesser et al. 2016). *Albidovulum* members have previously been found in marine and terrestrial hot springs (Albuquerque et al. 2002, Yin et al. 2012) suggesting that members of this genus are heat-tolerant. Other abundant ASVs common to all treatments were assigned to the Sva0996 group, PAUC26f group, and Rhodobacteraceae family, all of which have been frequently found in association with HMA sponges (Moitinho-Silva et al. 2017b, Turon et al. 2018).

In contrast to the above, HS increased the relative abundances of the BD2-11 terrestrial group, order Saccharimonadales and eight ASVs assigned to the BD2-11 terrestrial and HOC36 groups, SAR202 and KI89A clades, Rhodobacteraceae and Hyphomonadaceae families, and Pseudohongiella genus. All of these ASVs were closely related to bacteria previously detected in sponges (94%–100% sequence similarities, Table S8, Supporting Information). SAR202 members collected from the sponge *A. aerophoba* were suggested to degrade dissolved organic matter in seawater, aiding nutrient acquisition in their host sponges (Bayer et al. 2018). Sponge-associated Rhodobacteraceae displayed the genomic potential to reduce nitrogen-containing aromatic compounds and utilize sulphated polysaccharides, potentially contributing to benthic biogeochemical cycling (Karimi et al. 2019). Although the metabolic capabilities of BD2-11 terrestrial group members associated with sponges are not fully understood, members of this group have previously been found to decompose recalcitrant soil organic matter, such as HS, in soils (Pascault et al. 2013). Similarly, Saccharimonadales members have previously been found in soil, where they are thought to play a key role in phosphorus cycling by converting organic phosphorus to inorganic forms (Wang et al. 2022). It is possible that these bacterial groups were favored over other bacterial populations in the HS-supplemented microcosms due to their common ability to degrade complex organic molecules.

The relative abundances of the HOC36 group, orders Nitrosococcales, Dadabacteriales, Steroidobacterales, Puniceispirillales, and Thalassobaculales were negatively associated with HS. More specifically, we detected nine ASVs assigned to the HOC36 group, JTB255 marine benthic group (Steroidobacterales order), OM75 clade (Thalassobaculales order), TK17 group, Dadabacteriales order, Simkaniaceae family (Chlamydiales order), and the genera *AqS1* (Nitrosococcales order), *Constrictibacter* (Puniceispirillales order), and *Pseudohongiella* (Oceanospirillales order), which were negatively associated with HS. These bacterial groups are often detected in low nutrient environments (Rappé et al. 1997, Nelson et al. 2014, Graham and Tully 2021) with metabolic profiles specialized to obtain nutrients from inorganic sources. Nitrosococcales, e.g., includes marine bacterial ecotypes involved in the recycling of inorganic nitrogen (Semedo et al. 2021). Moreover, the gene repertoire of *AqS1* members detected in the sponge *Amphimedon queenslandica*, indicated that they were capable of sulfur oxidation, carbon monoxide oxidation, and inorganic phosphate assimilation (Gauthier et al. 2016). JTB255 members have frequently been found in marine sediments and metagenome analysis has suggested that they are involved in sulfur oxidation and carbon fixation (Mußmann et al. 2017, Hoffmann et al. 2020).

Previous studies have suggested that sponges play crucial roles in nutrient cycling and energy transfer within coral reefs (De Goeij et al. 2013, Alexander et al. 2014, Rix et al. 2016). Given the effect of HS on sponge-associated bacteria observed in the present study, it will be interesting to further explore the potential role of sponges in the transfer of nutrients obtained from terrestrially derived organic matter, and HS specifically, to the marine food-web.

Elevated temperature (Heat) positively influenced the relative abundances of the order Planctomycetales and two proteobacterial ASVs (8 and 29), assigned to the families Pseudohongiellaceae and Hyphomonadaceae. Note that this effect in Planctomycetales was seen in *Heat* and *Heat + HS*, but less so in *UVB + Heat* and *UVB + Heat + HS*. Additionally, ASV-29 was only detected in HS-treated microcosms and the effect of Heat, therefore, only holds true for *Heat + HS* and *UVB + Heat + HS*. Members of the order Planctomycetales have previously been found in association with both abiotic and biotic biotopes in terrestrial and aquatic environments (Wiegand et al. 2018). In aquatic environments, Planctomycetales members often associate with phototrophs (macro- and microalgae, and cyanobacteria) and have been shown to have the genomic potential to degrade algal-derived sulphate polysaccharides (Glöckner et al. 2003, Bengtsson et al. 2012, Wegner et al. 2013). Additionally, Planctomycetales members have been detected in various sponge species (Pimentel-Elardo et al. 2003, Mohamed et al. 2010, Kohn et al. 2020) and were particularly abundant in visually classified “abnormal” tissue of the sponge *Carteriospongia foliascens* (Gao et al. 2014), now known as *Phyllospongia foliascens*. ASV-8, assigned to the *Pseudohongiella* genus (Pseudohongiellaceae family), was recorded across all treatments but was significantly more abundant in heat-treated microcosms. Chaib De Mares et al. (2018) previously showed that close relatives of *Pseudohongiella* were among the most metabolically active bacterial associates of *A. aerophoba*. Monti et al. (2021), furthermore, observed them to be enriched in diseased tissue of *A. cauliformis*. Moreover, and in accordance with their specific proliferation in heat-treated microcosms, *Pseudohongiella* strains isolated from seawater grew optimally at a temperature of 30°C (Xu et al. 2016). ASV-29, assigned to the Hyphomonadaceae family, was only recorded in HS-treated microcosms, and was significantly more abundant in Heat + HS-supplemented microcosms. Hyphomonadaceae are aerobic, heterotrophic bacteria and have been found in host and environmental marine biotopes (Abraham and Rohde 2014, Podell et al. 2020). Podell et al. (2020) detected two Hyphomonadaceae members among metagenome assembled genomes obtained from the phototrophic sponge *Lamellodysidea herbacea*. Based on their genomic profile, these were suggested to degrade host-extracellular matrix, organic marine particulates, and aromatic compounds such as benzoic acid (Podell et al. 2020).

In contrast to the above, ASV-1739 (KI89A clade) was less abundant in *Heat* and *Heat + UVB* treated microcosms. Schellenberg et al. (2020) reported the specific loss of KI89A clade members associated with the sponge *Haliclona cnidata* following experimental exposure to detrimental conditions (light exclusion and addition of antibiotics). The relative abundances of KI89A clade members were also negatively affected by increasing temperature in the temperate sponge *A. oroides* (Castro-Fernández et al. 2023). In addition to their association with sponges, KI89A clade members are common oligotrophic marine bacteria that do not usually grow in nutrient-rich environments (Cho and Giovannoni 2004). Interestingly, our results showed that ASV-1739 was present in all *Heat + HS* and *Heat + UVB + HS* microcosms, which indicates HS might, to some extent, have mitigated the adverse effect of elevated temperature on this specific ASV.

## Conclusion

Here, we observed that *Chondrilla* sp. hosted a core bacterial community unaffected by experimental differences in temperature, UVB radiation or HS-supplementation, and consisting of members related to organisms obtained from other HMA sponges. However, *Chondrilla* sp. also hosted a variable bacterial community, affected by HS and, to a lesser extent, elevated temperature. Overall, both HS and elevated temperature significantly modified bacterial community composition and taxon abundance, whereas no interactive or UVB-related effects were observed. Elevated temperature positively affected the relative abundances of the order Planctomycetales and ASVs of the proteobacterial families Pseudohongiellaceae and Hyphomonadaceae, but negatively affected the abundance of a potential symbiont of the KI89A clade. HS, in turn, positively affected the relative abundances of several ASVs assigned to taxa potentially involved in recalcitrant organic matter degradation (e.g. the BD2-11 terrestrial group, Saccharimonadales, and SAR202 clade).

## Supplementary Material

fiae022_Supplemental_Files

## Data Availability

Sequences generated in this study can be downloaded from the NCBI Sequence Read Archive under the BioProject accession number PRJNA904682.
