# Supplementary material for: Impacts of humic substances, elevated temperature, and UVB radiation on bacterial communities of the marine sponge Chondrilla sp
Source: FEMS Microbiol Ecol. 2024 Feb 16;100(3):fiae022. doi: 10.1093/femsec/fiae022 (PMC10939426; doi:10.1093/femsec/fiae022)
Supplement: fiae022_Supplemental_Files [file fiae022_supplemental_files.zip › Supplementary_material_figures_REVISED2.pdf]

Supplementary material

Impacts of humic substances, elevated temperature and UVB radiation on bacterial communities of the marine sponge *Chondrilla* sp.

T.M. Stuij*, D.F.R. Cleary*, R.J.M. Rocha, A.R.M. Polonia, D.A.M. Silva, A. Louvado, N.J. de Voogd, N.C.M. Gomes*

Published in: FEMS microbiology ecology

*Corresponding authors:
tamarastuij@ua.pt (T.M. Stuij), cleary@ua.pt (D.F.R. Cleary) & gomesncm@ua.pt (N.C.M. Gomes)

Lighting system

Lighting was controlled by four fully programmable luminaire systems (Reef - SET, Rees, Germany), each holding eight fluorescent lamps 17. During the current experiment, four UV fluorescent tubes (SolarRaptor, T5/54W) and four full spectra fluorescent tubes (ATI AquaBlue Special, T5/54W) were connected alternately. To simulate photoperiod conditions of tropical latitudes, the lamps were programmed to a 12 h diurnal light cycle with light intensity varying from 2.61*10-3 J cm-2 s-1 in the morning to 8.82*10-3 J cm-2 s-1 at mid-day (measured using a Fibre optic probe positioned at the water surface, Flame spectrometer, Ocean Optics). The total light energy transmitted during the day equalled 256 J cm-2 day-1, of which 97.7% came from the photoactive radiation (PAR) wavelengths (300 – 700), 1.85% from the UVA wavelengths (315 – 400) and 0.43% from the UVB wavelengths (280 – 315). Photosynthetic photon flux density (PPFD, mol photons m-2 s-1) at the sediment-water interface was measured using an underwater PAR meter (MQ-510 PAR/Quantum-Meter Underwater, Apogee, USA). A total of 3.47 ± 0.68 mol photons m-2 d-1 reached the sediment surface. A transparent polyester film (Folanorm SF-AS, Folex coating, Köln, Germany) was used to block UVB light (290 – 320 nm) 87,88. This film absorbed 90% of the UVB irradiance, 31% of UVA and 9% of PAR irradiance. A detailed figure of the light spectrum can be found in Supplementary Fig. 2.

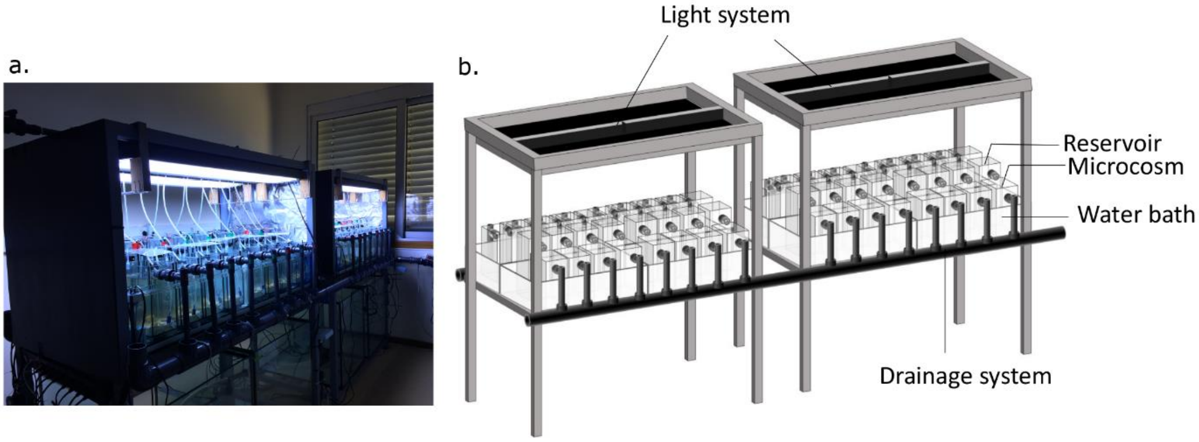

Supplementary Fig. 1 Picture (a) and graphical representation (b) of the experimental life support system.

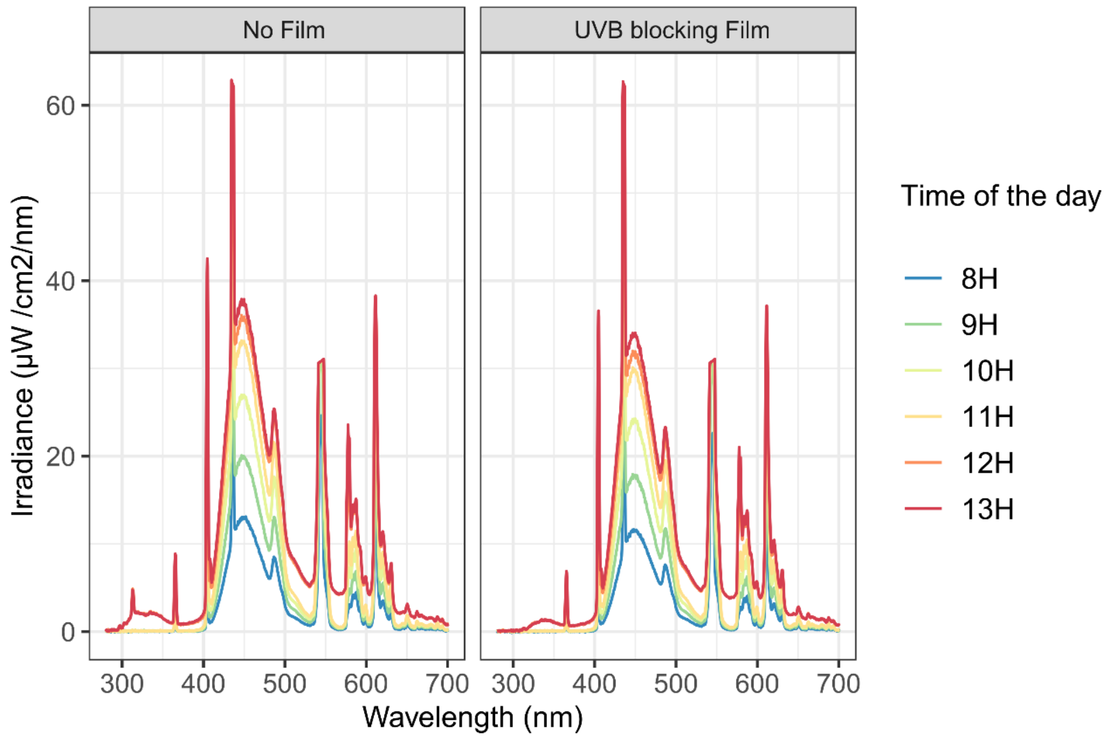

Supplementary Fig. 2 Irradiance spectrum of the fluorescent lamps measured at the point where the light would hit the water surface with and without the transparent polyester film (Folanorm SF-AS, Folex coating, Köln, Germany). Light intensity increased from 8.00 in the morning to 13.00 in the afternoon.

Source code of the function adonis_OmegaSq()

```
adonis_OmegaSq <- function(adonisOutput, partial = TRUE){
   if(!(is(adonisOutput, "adonis") || is(adonisOutput, "anova.cca")))
      stop("Input should be an adonis object")
   if (is(adonisOutput, "anova.cca")) {
      aov_tab <- adonisOutput
      aov_tab$MeanSqs <- aov_tab$SumOfSqs / aov_tab$Df
      aov_tab$MeanSqs[length(aov_tab$Df)] <- NA
   } else {
      aov_tab <- adonisOutput$aov.tab
   }
   heading <- attr(aov_tab, "heading")
   MS_res <- aov_tab[pmatch("Residual", rownames(aov_tab)), "MeanSqs"]
   SS_tot <- aov_tab[rownames(aov_tab) == "Total", "SumsOfSqs"]
   N <- aov_tab[rownames(aov_tab) == "Total", "Df"] + 1
   if(partial){
      omega <- apply(aov_tab, 1, function(x) (x["Df"]*(x["MeanSqs"]-MS_res))/(x["Df"]*x["MeanSqs"]+(N-
x["Df"])*MS_res))
      aov_tab$parOmegaSq <- c(omega[1:(length(omega)-2)], NA, NA)
   } else {
      omega <- apply(aov_tab, 1, function(x) (x["SumsOfSqs"]-x["Df"]*MS_res)/(SS_tot+MS_res))
      aov_tab$OmegaSq <- c(omega[1:(length(omega)-2)], NA, NA)
   }
   if (is(adonisOutput, "adonis"))
      cn_order <- c("Df", "SumsOfSqs", "MeanSqs", "F.Model", "R2",
               if (partial) "parOmegaSq" else "OmegaSq", "Pr(>F)")
   else
      cn_order <- c("Df", "SumOfSqs", "F", if (partial) "parOmegaSq" else "OmegaSq",
               "Pr(>F)")
   aov_tab <- aov_tab[, cn_order]
   attr(aov_tab, "names") <- cn_order
   attr(aov_tab, "heading") <- heading
   if (is(adonisOutput, "adonis"))
      adonisOutput$aov.tab <- aov_tab
   else
      adonisOutput <- aov_tab
   return(adonisOutput)
}
```

Water quality analysis

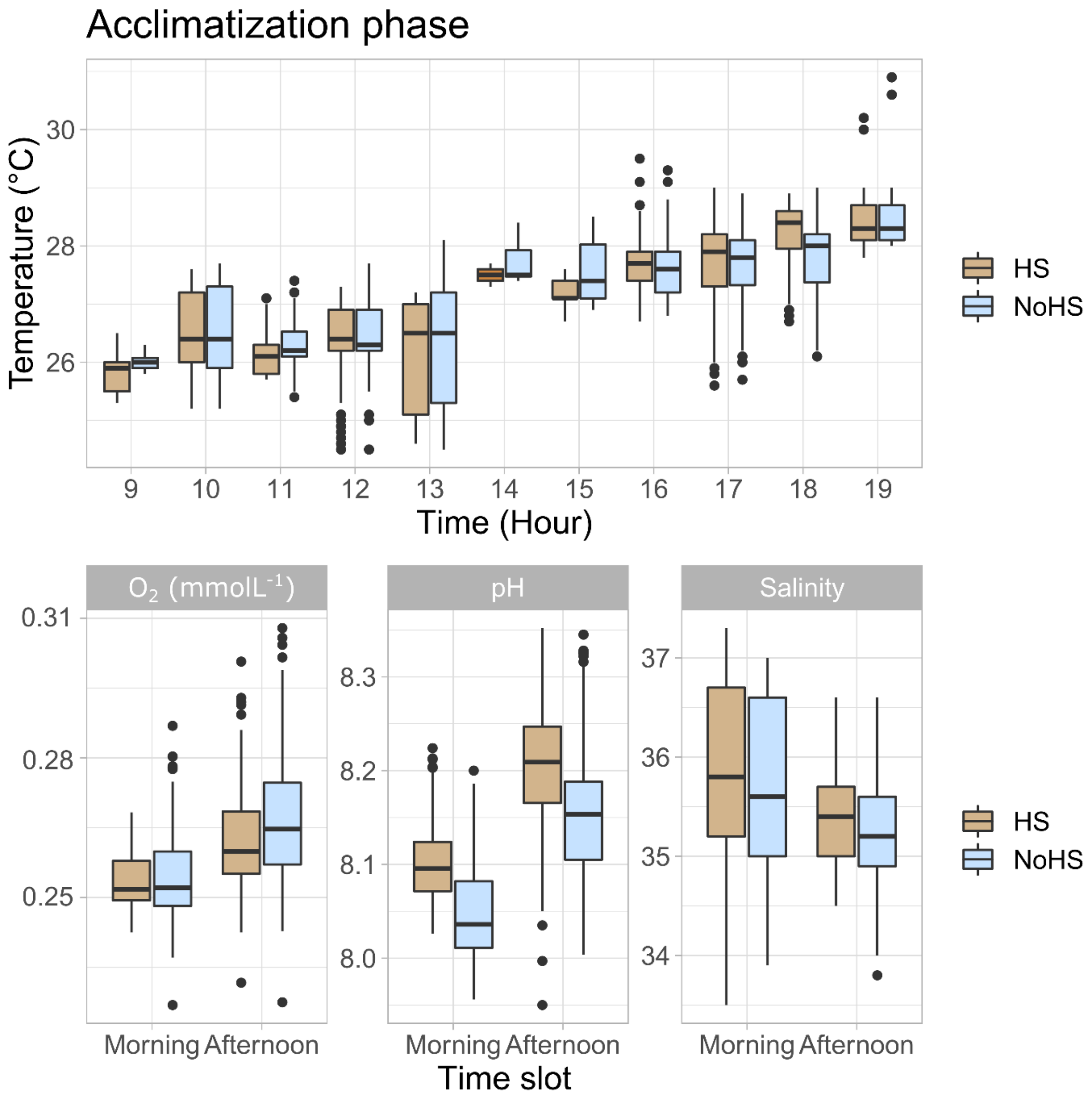

Supplementary Fig. 3 Temperature (hourly), oxygen, pH and salinity (two times per day) during the acclimatization phase of the experiment (21 days).

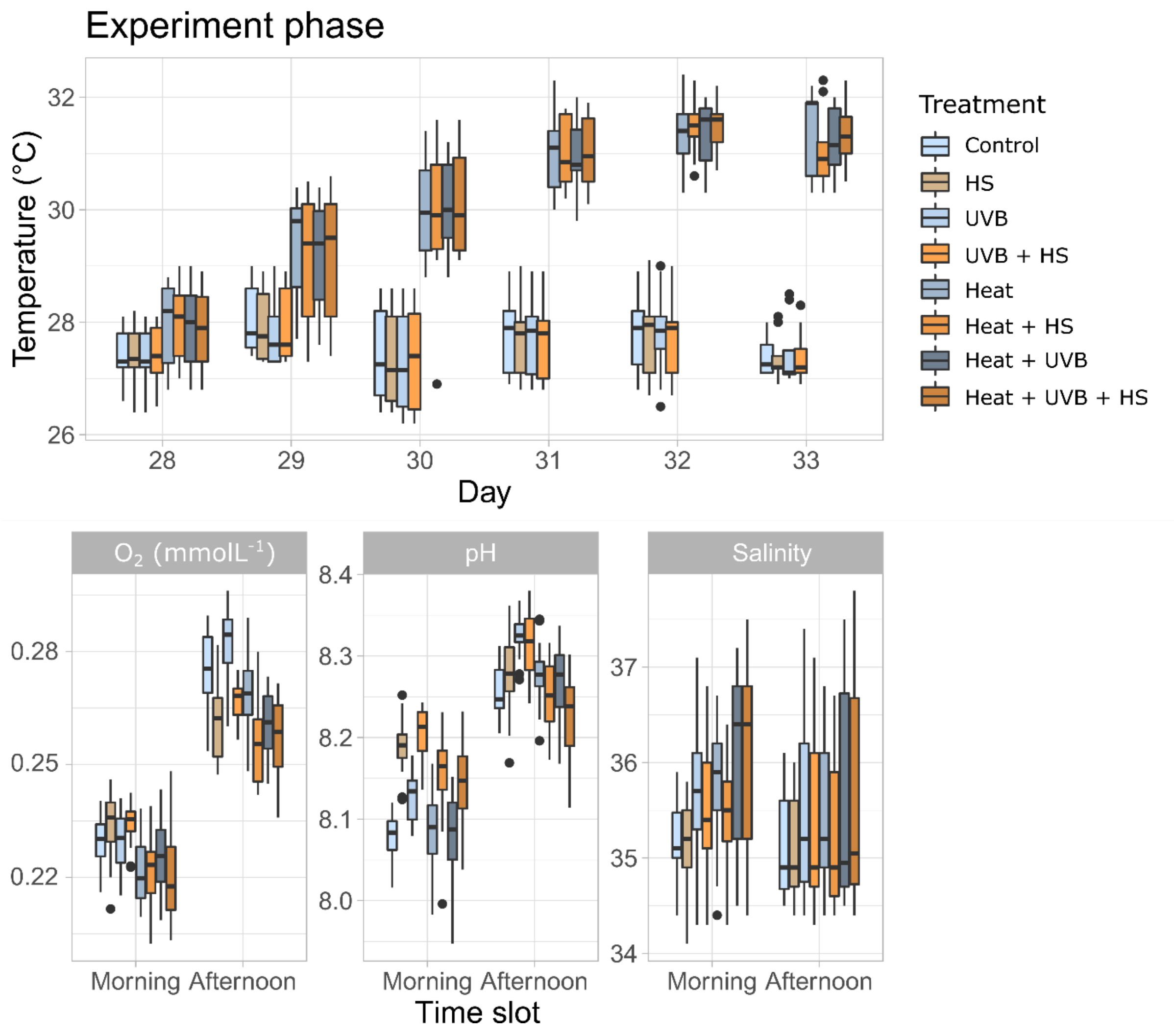

Supplementary Fig. 4 Temperature (daily measurement between 8:00 and 9:00 am), oxygen, pH and salinity (two times per day) during the experiment phase of the experiment (5 days).

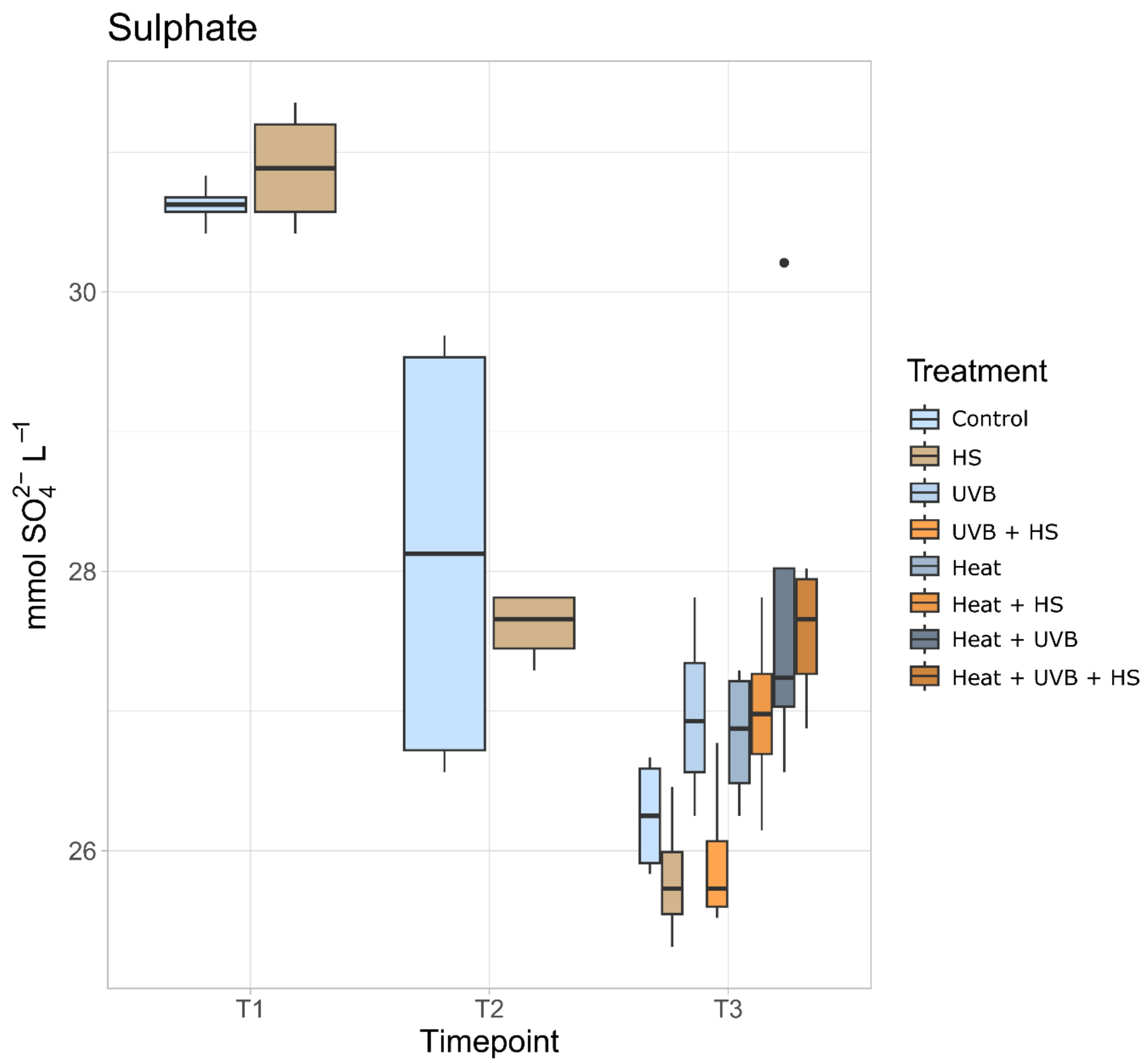

Supplementary Fig. 5 Sulphate (SO_4^{2-}) concentration in the sediment pore water. Day 8) measurement right before addition of the reef organisms, day 29) measurement right before exposure to heat and UVB treatments, day 34) end of the experiment.

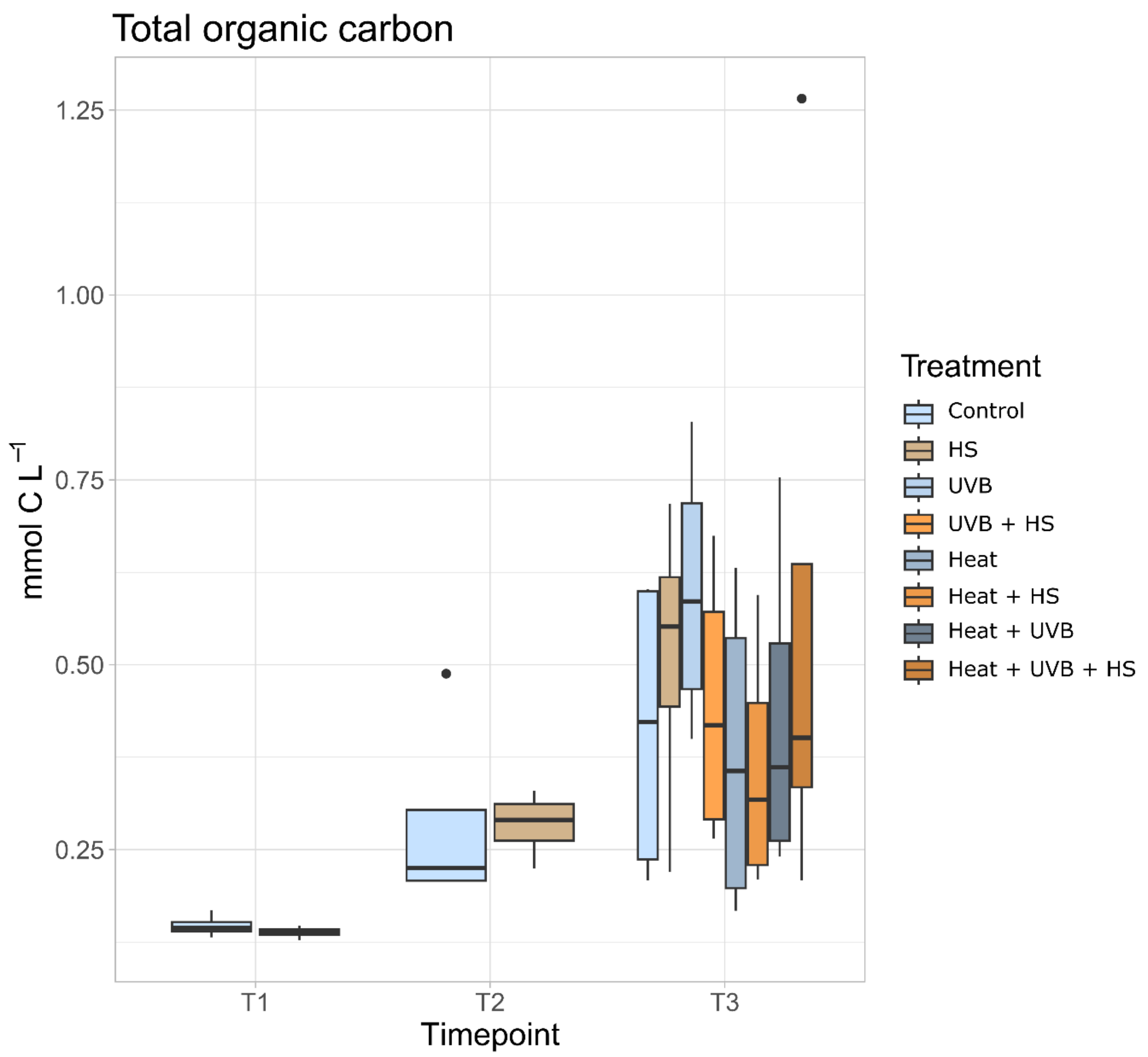

Supplementary Fig. 6 Total organic carbon concentration in the sediment pore water measured using Rhizon flex samplers with a pore size of 0.6 µm (product number 19.60.25F, Rhizosphere Research Products). Day 8) measurement right before addition of the reef organisms, day 29) measurement right before exposure to heat and UVB treatments, day 34) end of the experiment.

Bacterial community composition in ECOMARE and transplantation effect

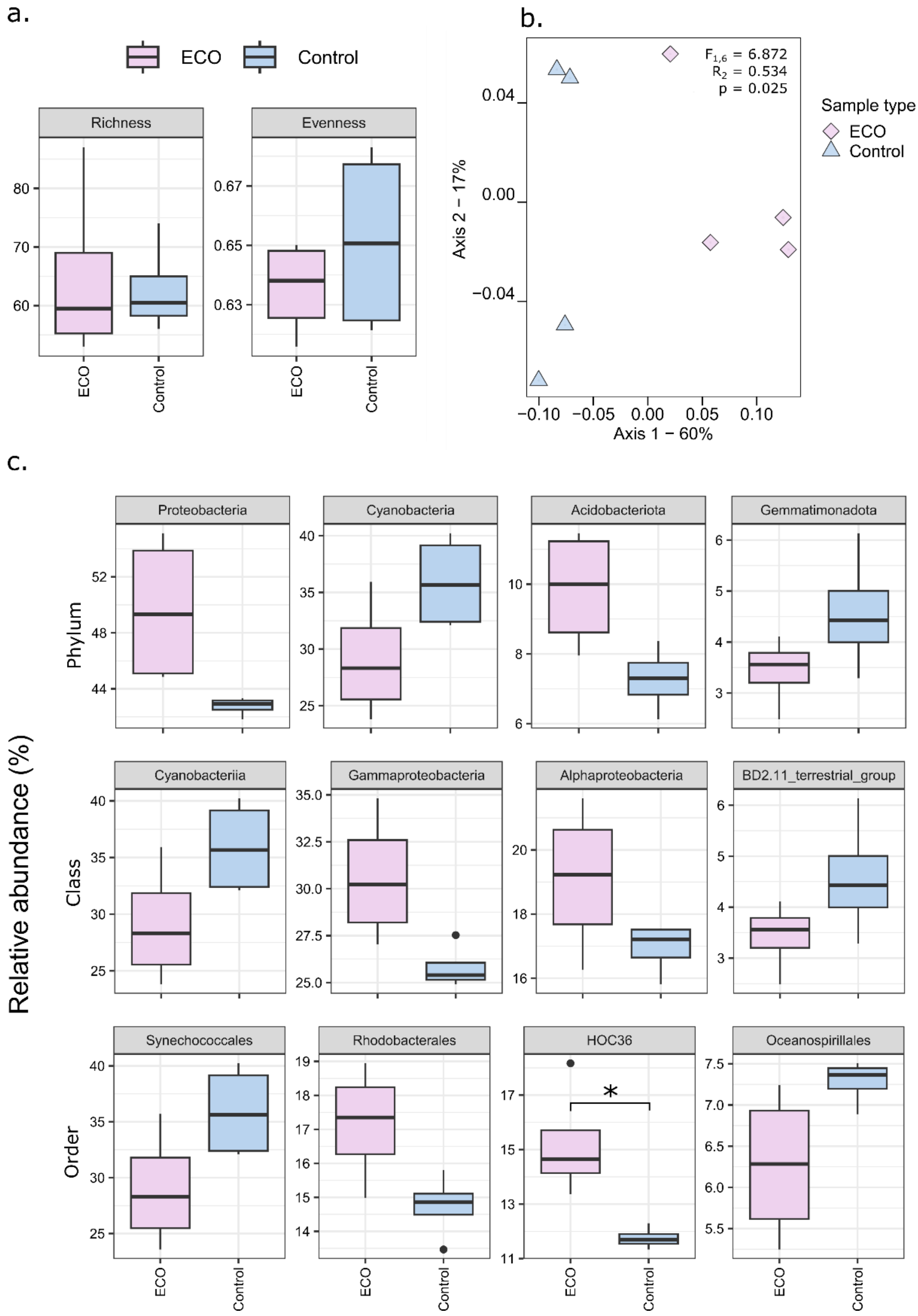

Supplementary Fig. 7 Diversity analysis comparing samples taken from ECOMARE and microcosm controls. a) Boxplots of rarefied bacterial richness and evenness, b) Ordination showing the first two axes of the principal coordinates analysis (PCoA) of bacterial OTU composition. Results of the PERMANOVA (performed using the adonis() function in the R package *vegan*) are depicted in the upper right corner. c) Boxplots of the relative abundance of the four most abundant phyla, classes and orders. Significant differences (GLM, $p < 0.0125$, see table S5) in higher taxonomic abundance are indicated with an * (HOC36).

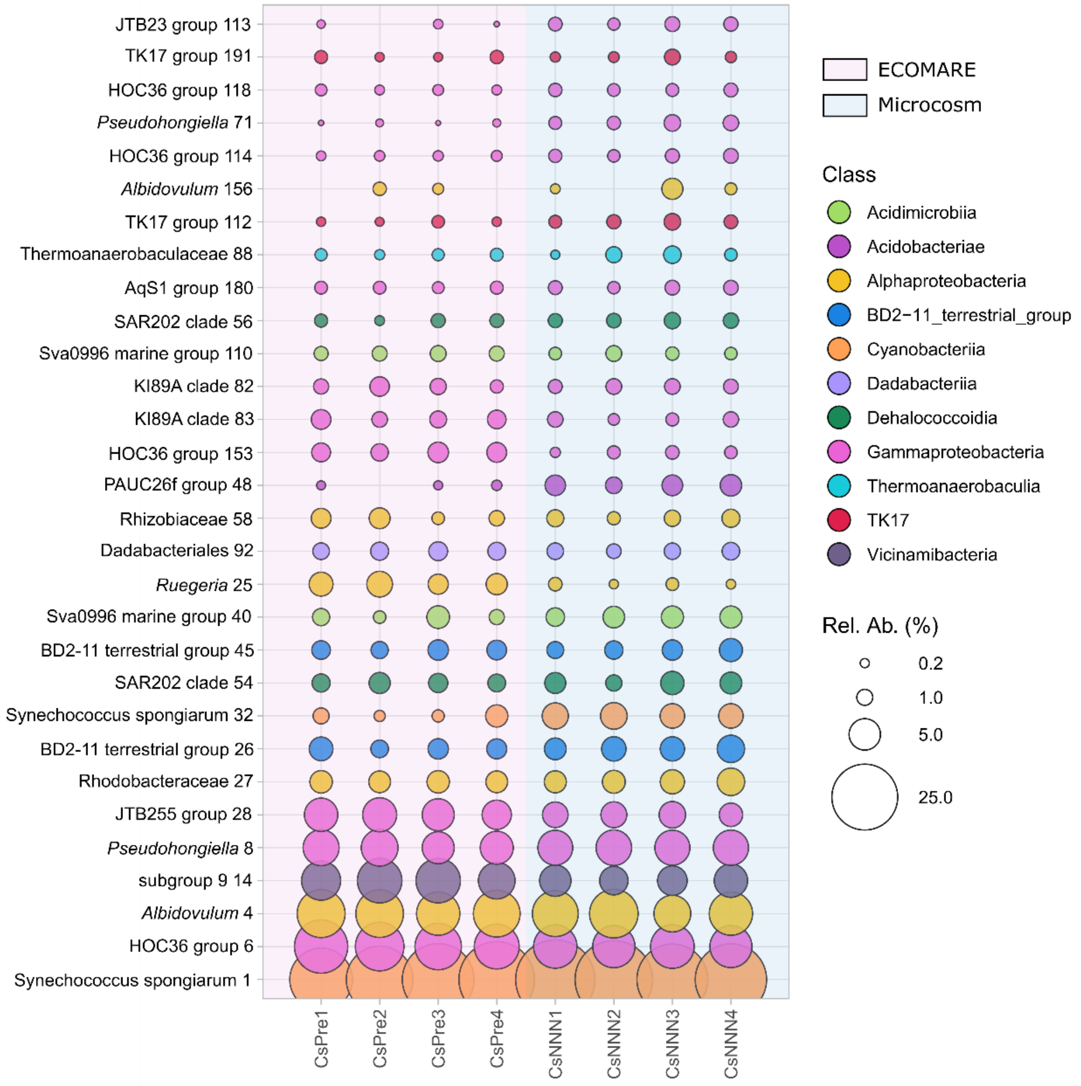

Supplementary Fig. 8 Mean relative abundances of the 30 most abundant ASVs in the sponges collected from ECOMARE (CsPre) and Control (CsNNN) microcosms. ASVs are labelled with their respective number and lowest taxonomic assignment. Symbols are proportional to the relative abundance of the respective OTU and colour-coded following their class-level taxonomic assignment.
